# Bariatric emergencies: current evidence and strategies of management

**DOI:** 10.1186/1749-7922-8-58

**Published:** 2013-12-29

**Authors:** Abdulzahra Hussain, Shamsi EL-Hasani

**Affiliations:** 1 Minimal access and bariatric unit, King’s College Hospital NHS Foundation Trust, Princess Royal University Hospital, Orpington, London BR6 8ND, UK; 2 Honorary Senior Lecturer at King’s College Medical School, London, UK

**Keywords:** Laparoscopic roux en-Y gastric bypass, Laparoscopic sleeve gastrectomy, Laparoscopic adjustable gastric band, Stomal ulceration

## Abstract

**Background:**

The demand for bariatric surgery is increasing and the postoperative complications are seen more frequently. The aim of this paper is to review the current outcomes of bariatric surgery emergencies and to formulate a pathway of safe management.

**Methods:**

The PubMed and Google search for English literatures relevant to emergencies of bariatric surgery was made, 6358 articles were found and 90 papers were selected based on relevance, power of the study, recent papers and laparoscopic workload. The pooled data was collected from these articles that were addressing the complications and emergency treatment of bariatric patients. 830,998 patients were included in this review.

**Results:**

Bariatric emergencies were increasingly seen in the Accident and Emergency departments, the serious outcomes were reported following complex operations like gastric bypass but also after gastric band and the causes were technical errors, suboptimal evaluation, failure of effective communication with bariatric teams who performed the initial operation, patients factors, and delay in the presentation. The mortality ranged from 0.14%-2.2% and increased for revisional surgery to 6.5% (p = 0.002). Inspite of this, mortality following bariatric surgery is still less than that of control group of obese patients (p = value 0.01).

**Conclusions:**

Most mortality and catastrophic outcomes following bariatric surgery are preventable. The awareness of bariatric emergencies and its effective management are the gold standards for best outcomes. An algorithm is suggested and needs further evaluation.

## Introduction

### Definitions

Bariatric surgery: Relating to the treatment of obesity. Origin: Greek bar (os) weight (cf. baro-) + −iatrics (http://dictionary.reference.com/browse/bariatrics).

British Obesity and Metabolic Surgery Society BOMSS defined it as: Surgeons involved in obesity management (http://www.bomss.org.uk).

Metabolic surgery: The procedures for weight loss, whether designated as restrictive, restrictive/malabsorptive, malabsorptive, and others, or neuro-hormonal, all fall under the definition as the operative manipulation of a normal organ or organ system to achieve a biological result for a potential health gain [1].

### Current indications for bariatric surgery

The National Institute for health and Clinical Excellence (NICE) UK guidelines and the American Gastroenterological Association (AGA) recommend bariatric surgery as a treatment option for adults with a Body Mass Index (BMI) 40–50, or those with BMI > 35-40 and one or more severe obesity-related medical complication (e.g., hypertension, heart failure, or sleep apnea) if they have been unable to achieve or maintain weight loss with conventional therapy, have acceptable operative risks, and are able to comply with long-term treatment and follow-up [2,3]. The updates of these indications are going to be published in the Endocrine Practice 2013; the most updated version of guidelines sponsored by American Association of Clinical Endocrinologists (AACE), The Obesity Society (TOS), and American Society for Metabolic & Bariatric Surgery (ASMBS).

Types of operations: Different types of operations including gastric balloon, gastric plication and different types of intestinal bypasses are practiced but the most popular are: Gastric bypass, Sleeve gastrectomy, Gastric band, Bilio-pancreatic diversion+/−Duodenal switch.

### Background

Bariatric and metabolic surgery to treat obesity and type 2 diabetes has promising results of cure rather than improvement [4], Level of evidence (L2+), such outcome is not without risks due to a limited physiological reserve, thus patients can easily deteriorate when complications occur. Modern technology and minimal access experience have contributed to the current quality and safety of bariatric operations but unfortunately mortality is still reported in the literature and especially after complex procedures such as duodenal switch and bilio-pancreatic diversion and to a less extent after gastric bypass [5,6], (L2+). The Swedish Obese Subject Study has shown clear benefits of Bariatric Surgery (BS) in terms of reduced mortality and weight loss compared to control group of conventional therapy [7]. Such benefits can be improved by reducing the mortality of emergencies of BS.

The AACE, TOS and ASMBS updated document of guidelines is referring to 6 recommendations concerning acute presentations of the bariatric patients, stressing the admission and surgical approach if medical treatment is not effective for malnutrition, gastrointestinal complications, strictures and revisional surgery [8], (variable levels). Currently there are no comprehensive guidelines for managing the acute presentations of BS and the on-call general surgical and anaesthetic/critical care teams are increasingly facing such clinical scenarios with no standard plan of management.

To address the problem of morbidity and mortality, it is important to predict which patient is going to develop complications after BS and one way of doing so is by using certain indices; like the Elixhauser index [9], (L2+).

Recent study of more than 44,000 patients from the United States of America (USA) showed independent predictors associated with significantly increased mortality included age > 45 years, male gender, a body mass index (BMI) of 50 kg/m or higher, open bariatric procedures, diabetes, functional status of total dependency before surgery, prior coronary intervention, dyspnea at preoperative evaluation, more than 10% unintentional weight loss in 6 months, and bleeding disorder [10,11], (L2+). Intensive care unit admission for bariatric patients has increased owing to revisional surgeries [12], (L2+), and the trend has changed from elective to an emergency admission.

The Accident & Emergency (A&E) doctors, the on-call surgical teams and sometimes the on-call medical team are initially seeing and managing bariatric patients for different presentations [13] (L2+), one of the reasons of poor outcomes of these patients is primary failure of recognition of the problem [14], (L2++). Hence, there is a definite need for clear pathways to reduce the incidents of delaying investigations, initiating appropriate treatment and start effective communication with bariatric and critical care teams [15], (L2+), [16,17], (L2++). The aim of this document is to study the current evidence and to highlight the initial management plan for emergencies of BS and streamlining a timely and safe management pathway.

## Materials and methods

The PubMed and Google engines were searched for English literatures relevant to the acute presentations and management of bariatric emergencies. The search words were bariatric emergencies, management of acute bariatric patients, acute abdomen in obesity and metabolic surgery; post bariatric complications, nutritional complications following bariatric and metabolic surgery.Two independent authors selected the studies. 6358 articles were found. The abstracts of these articles were filtered and 90 papers were selected for this review. The pooled data from selected studies were further analysed. Heterogeneity was obvious. A review was undertaken to assess the evidence in each of 7 common complications (bleeding, leak, obstruction, stomal ulceration, pulmonary embolism and respiratory complication, blood sugar disturbances and nutritional disturbances) that were seen in the A&E department and required an urgent and safe approach.

Scottish Intercollegiate Guidelines Network (SIGN) grading of evidence was adopted (see Table 1). The heterogeneity among the studies because of the different causes of morbidity and mortality after different types of BS for different categories of patients and different surgical teams and centres, has made a robust meta-analysis or systematic review almost impossible. The reference followed by the level of evidence (L) was used for citation.

**Table 1:** Level of evidence

**Levels of Evidence (Scottish Intercollegiate Guidelines Network, SIGN)**	
1++	High quality meta-analyses, systematic reviews of RCTs, or RCTs with a very low risk of bias
1+	Well conducted meta-analyses, systematic reviews, or RCTs with a low risk of bias
1-	Meta-analyses, systematic reviews, or RCTs with a high risk of bias
2++	High quality systematic reviews of case control or cohort studies
	High quality case control or cohort studies with a very low risk of confounding or bias and a high probability that the relationship is causal
2+	Well conducted case control or cohort studies with a low risk of confounding or bias and a moderate probability that the relationship is causal
2-	Case control or cohort studies with a high risk of confounding or bias and a significant risk that the relationship is not causal
3	Non-analytic studies, eg case reports, case series
4	Expert opinion

## Results

**Figure 1:**
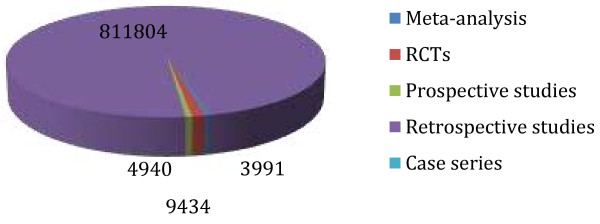
Weight of the studies.

**Figure 2:**
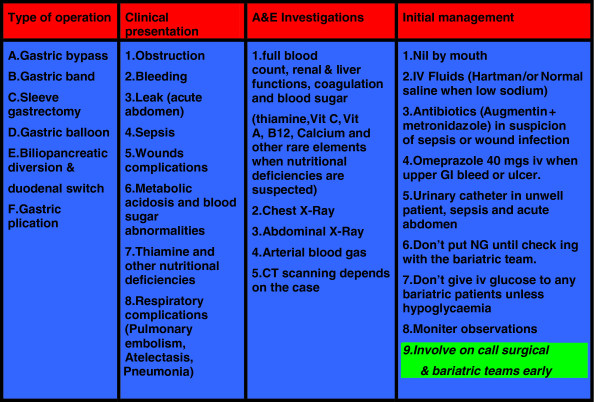
Acute presentations of bariatric surgeries and initial accident & emergency management.

**Figure 3:**
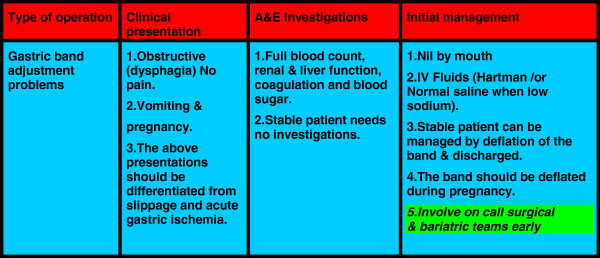
Acute presentations and management of gastric band adjustment problems.

**Figure 4:**
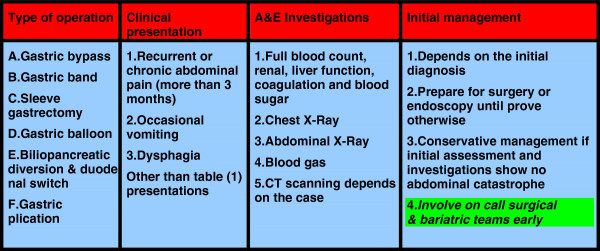
Presentations and initial management of chronic or subacute post bariatric surgery complications which are seen in emergency department.

The pooled data of 830,998 patients were included in this study. The evidence was variable in strength [4 guidelines, one meta-analysis, 10 Randomised Controlled Trials (RCTs), 2 comparative studies, 4 prospective cohorts, 20 reviews, 48 retrospective observational studies and 9 case series (in rare complications only)]. Majority of the current body of knowledge was reported by retrospective and observational studies (see Figure 1). 80% of the presented evidence was published in the last 5 years. Catastrophic and avoidable mortality and severe disabling morbidities were still seen in the current bariatric practice, complex multifactor aetiology was reported and included low degree of awareness among the admitting teams, unavailability of bariatric services at the admitting hospitals, failure of communication with the bariatric team who performed the initial operation, technical errors and delay in the presentation and referral by general practitioners, and personal factors. The outcomes of each of research areas were reported under each of the following headings. As a result a conclusion algorithm was suggested (see Figures 2, 3 and 4).

### Bleeding

Bleeding complications could follow any operation and bariatric surgery is not an exempt. Luckily, relatively rare, following gastric bypass it was around 0.94% [18], (L2+), but it could reach 4.4% [19], (L2+). A systematic review concluded a higher rate in laparoscopic Roux en-Y Gastric bypass (LRYGB) versus open one [20], (L2+). Bleeding following Adjustable Gastric Band (LAGB) was extremely rare and could be related to organ injury or band erosions [21], (L3), [22], (L2+). Bleeding following LRYGB, sleeve gastrectomy and bilio-pancreatic diversion could be intralumenal or extralumenal types. Early bleeding (within 30 days of operation) was more common than late bleeding. The presentation was variable and diverse from low haemoglobin to fainting and un-stability, heamatemesis, malena and fresh blood per rectum were another forms [23], (L3), small bowel obstruction may be the initial presentation of bleeding and can occur in 0.5% of patients [24], (L2+), [25], (L2++).

The underlying causes of bleeding could be due to patient factors such as bleeding tendencies, anti-coagulations, infection, ulceration or surgeon factors of poor surgical or haemostatic technique, while the type and the length of the operation is reflected by the degree of complexity (the more complex and prolong procedure, the more the incidence of bleeding) [26], (L2++). LRYGB caused more bleeding complication [27], (L2++), which can be related to the type of stapler. A meta-analysis showed linear stapler was associated with less bleeding [28], (L1+), and enforcement of staple line after Laparoscopic Sleeve Gastrectomy (SG) could reduce bleeding significantly (odd ratio was 0.559) [29], (L2++).

NICE guideline of acute upper gastrointestinal bleeding management was an excellent reference [30], (variable levels). However, some specific measures may help to control the bleeding in this particular group of patients [31], (L2+), [32], (L3). This was largely related to the type of the procedure and whether the bleeding was intra or extra luminal. Intra-lumenal bleeding especially at the gastro-jejunostomy (G-J) can be controlled with the aid of endoscopic therapy [33], (L2++). Jejuno-jenunostomy (J-J) intra lumenal bleeding site may be difficult to reach with good visualization by the endoscopy and majority of unstable patients were candidates of laparoscopic or laparotomy exploration of the anastomosis and controlling of bleeder(s). Extra-lumenal bleeding in stable patient can be treated conservatively. Unstable cases were managed by laparoscopic or laparotomy exploration [18], (L2+), with the use of clips, diathermy, suturing of bleeding points if the cause was staple line or more aggressive approach of splenectomy if the source was uncontrollable splenic bleed due to organ injury. Mesenteric, port site bleeder and other intra peritoneal bleeders can be controlled accordingly. As some of the patients would need blood transfusion for the bleeding, this raised the question of safety of bariatric surgery in 0.5% of American patients who refused transfusion for religious or personal causes. Safety was concluded for these patients, provided other measures have been used to manage the bleeding [34], (L2+).

### Leak

Leak was a major killer and an enemy to the success of BS, it can follow procedures where anastomosis is undertaken like gastric bypass, the incidence of which could reach 1.4% [35], (L2+). It can also follow laparoscopic Sleeve Gastrectomy (SG) or organ injury during LAGB insertion. Sepsis was reported as a major cause of death and this was largely due to diagnosed or undiagnosed leak [36], (L2+). After gastric bypass, a large study of 226,452 patients showed factors associated with higher risk of gastro-intestinal tract (GIT) leak were open gastric bypass, congestive heart failure, chronic renal failure, age older than 50 years, Medicare payer, male sex and chronic lung disease [37], (L2+). Routine use of per-operative endoscopy during LRYGB was associated with low leak (0.2%) and stricture (1.1%) rates [38], (L2+) while the use of drain provided no benefit and the diagnosis of leak was largely clinical, aided by appropriate imaging [39], (L2++). Leak was the most common cause of major morbidity and mortality after LSG [40], (L2+) and was associated with mortality rate of 4.5% [41], (L3).

Although reinforcement of sleeve line was thought to reduce the leak incidence [29], (L2++), a recent meta-analysis has failed to confirm that, but concluded the use of bougie larger than 40 FR was reducing the leak rate [42], (L2+), this may refer to the stenosis of the sleeve as a predisposing factor for leak in the LSG patients. The initial management of the leaks was that of peritonitis, some additional steps and tips depended on the type of operation. The choice of conservative or emergency exploration was dictated by the overall assessment of the patient. In a stable patient, the use of covered stent was effective [43], (L3). Emergency management was composed of drainage, control of sepsis, antibiotics, nutritional support [44], and intensive care management (L2++). If no response to the conservative treatment or deterioration, laparoscopy and/or laparotomy were suggested [45], (L3).

### Obstruction

Obstruction following bariatric surgery can be serious and GIT ischemia should be ruled out early in the course of presentation, late diagnosis and intervention could be disastrous [35], (L2+). Four types of internal hernia were seen following gastric bypass; Mesenteric, and mesojejunal, Petersons and jejuno-jejunal herniae are identified [46,47], (L2+), [48], (L2++). Obstruction could be due to a mechanical problem or technical error at the anastomosis or from banded gastric bypass operation [49], (L3). G-J stricture was another cause of obstruction and the incidence could reach 27% [50], (L2++). However, recent series reported much lower incidence of less than 1% [51], (L2+). J-J obstruction was reported and its incidence was much less that G-J stricture at 0.001%, the causes of which were different; technical error or clot obstruction [52,53], (L2+). Gastric band obstruction (a common complication) can be easily managed by deflation of the band. More serious slippage with gastric strangulation should be excluded in any case with dysphagia [54], (L2++). The initial assessment is outlined in the algorithm. The main question to the surgeon is: does this patient have gastrointestinal ischemia or not? Clinical presentation, Computerized Tomography (CT) scan, arterial blood gasses will help to direct the course of management.

### Stomal ulceration (SU)

This was an unusual complication following LRYGB. Early series reported high incidence of 12.5% [55], (L2+), however later studies showed much lower incidence of refractory SU of 0.1% [56], (L2+). Early experience showed SU was the result of acid production in the bypassed stomach in the presence of a gastrogastric fistula, could happen in 20% of patients [57], (L2+), however wider experience indicated that SU was caused by possible ischemia, narrow G-J stoma, smoking, medications including steroid and Non Steroidal Anti-Inflammatory Drugs (NSAID), H Pylori infection, foreign bodies at the base of ulcer [58], (L3), [59,60], (L2+).

Prophylactic short course of proton pump inhibitor PPI has reduced the incidence of SU [61], (L2+). The PPI may works in presence of Helicobacter Pylori infection. There are other studies that contradict these findings. The presentation of SU was variable, but ranged from epigastric pain to dysphagia, vomiting and obstruction. However, 28% were asymptomatic [62], (L2++).

Avoidance of the above risk factors should reduce the incidence of the SU. The management of established case was ranging from PPI and cytoprotective scrulfate, endoscopic and radiological dilatation of associated stricture and revisional surgery [63,64], (L2+).

### Pulmonary embolism and respiratory complications

Pulmonary embolism was rare after bariatric surgery; the weighted incidence was 0.5% [65], (L1+). The analysis of mortality of 13,871 patients in Italy after bariatric surgery showed 11.8% were due to respiratory failure including pulmonary embolism [66], (L2+). Not only LRYGB, but also LAGB was associated with major respiratory complications of aspiration pneumonia, atelectasis, exacerbation of asthma and empyema and lung abscess [67], (L2+). Prediction of patients who are likely to develop respiratory complication and prevention was the corner stone in reducing this kind of complications. Congestive heart failure and stroke were the greatest preoperative risk factors for pneumonia. Previous percutaneous coronary intervention, dyspnoea at rest, bleeding disorder, age, chronic obstructive pulmonary disease, and type of surgery, smoking, diabetes mellitus, anesthesia time and increasing weight were predisposing to respiratory complications [68], (L2+). Age, waist circumference, systolic blood pressure, and witnessed sleep apnea episodes were important risk factors in patient with sleep apnoea, thus preoperative optimization was necessary to reduce respiratory morbidity [69], (L2+). The main task for admitting and managing teams was to exclude abdominal catastrophe. When that’s done; the respiratory complications management was following the same concept of treatment of individual complication. Please see the algorithm (Figures 2, 3 and 4).

### Blood sugar disturbance

The American Diabetes Association (ADA) has defined the cure of diabetes following bariatric surgery as a return to normal measures of glucose metabolism (haemoglobin) HbA1c below 6 per cent, fasting glucose less than 5 · 6 mmol/l at least 1 year after bariatric surgery without hypoglycaemic medication [70], (L2+). All bariatric surgeries were influencing blood sugar level and improving insulin sensitivity and decreasing HgbA1c [71], (L2++), this effect was especially associated with more complex type such as gastric bypass, ileal or jejunal bypass and duodenal switch or bilio-pancreatic diversion [72,73], (L2++, [74], (L3). Hypotheses of the mechanisms were ranged from beta cell expansion to altered beta cell function as well as non-beta cell factors [75], (L2+). Post-prandial hyper insulinemic hypoglycemia associated with nesidioblastosis may be related to the changes in Glucagon Like Peptide (GLP-1) and other gut hormones [76], (L2++). The cases of hypoglycaemia and acidosis were seen in A&E department and can be successfully managed by joint care with the medical and endocrinology teams. Low carbohydrate diets may be effective in treating post-gastric bypass hyperinsulinemic hypoglycemia [77], (L3). Blood sugar disturbance and acidosis was also presented following LAGB and special association with early pregnancy was noticed. Deflation of the band will resolve the problem.

### Nutritional disturbances

Hypovitaminosis D and secondary hyperparathyroidism were associated with morbid obesity and therefore calcium deficiencies and acute hypocalcemia syndromes were reported following BS [78], (L2++), especially in patients who had thyroidectomy before [79], (L2++). 25-hydroxyvitamin D was the most commonly observed deficiency after SG & LRYGB [80], (L2++) while the major macronutrient deficiency after bariatric surgery was protein malnutrition [81], (L2++). A number of gastrointestinal or extra-gastrointestinal symptoms had raised the suspicion of malabsorption or dumping syndromes. Although it was rare for these patients to present acutely, physicians who care for patients after bariatric surgery need to be familiar with common postoperative syndromes that result from specific nutrient deficiencies [82,83], (L2++). Fat soluble vitamines and minerals abnormalities were reported following metabolic and bariatric surgery, the most common: vitamin B12, folate, zinc, thiamin, copper, vitamin A, and vitamin E deficiencies [84,85], (L2++), [86], (L1+), [87], (L2+), [88], (L2++). Water-soluble vitamins deficiency such as vitamin C (Scurvey) was extremely rare [89], (L3). Acute Wernicke’s encephalopathy secondary to thiamine and B12 deficiencies can induce permanent damage despite aggressive replacement therapy [90], (L3).

Acute presentation of these clinical syndromes was rare but great index of suspicion by General Practitioners (GPs) and bariatric teams is expected and needed for early diagnosis and effective treatment. In any acute presentation of the nutritional syndromes, acute surgical catastrophe has to be excluded first, then a multidisciplinary team approach of collaboration of admitting surgeon or physician, endocrinologist and gastroenterologist would resolve the problem. Acute psychiatric and neurological bariatric emergencies such as Wernicke’s encephalopathy would entail involvement of neuro-psychiatric teams.

### Algorithm of management of acute presentations after bariatric surgery

Based on the evidence from 90 papers including 830,998 patients an algorithm was suggested (Figures 2, 3 and 4).

This pathway is helping to initiate a correct management plan and to provide a standardised care for these critical patients. This algorithm is expected to induce hot discussions among surgeons, emergency physicians and endocrinologists, as there are no comprehensive guidelines of management. The current evidence of more than 800,000 patients is supporting the pathway and the conclusions of this article. The algorithm is in its initial stage and it needs independent assessment by further research.

### Limitations of the study

1. The opinions and conclusions of this review were based on the assessing pool data of the outcomes following bariatric operations. The presented evidence was variable in strength as in any meta-analysis or systematic review studies. Most recent studies were included. 80% studies were published in or after 2008. This supports the current evidence of how to manage this special group of patients.

2. The evidence was concluded from the most common operations; i.e. LRYGB, SG & LAGB. Referral to other operations like bilio-pancreatic diversion or duodenal switch was made on specific issues.

3. Great heterogeneity was found among the studies and a systematic review or meta-analysis looking at 7 complications following different bariatric operations, surgical teams and experience, type of patients, co-morbidities, period of follow up was considered impossible.

4. Studies selection: the abstracts of 6358 studies were assessed, only 90 studies were selected. Studies were excluded when they were small (less than 5 subjects, except in case of very rare complications like Scurvy following BS), not relevant to the research subjects. Some of the studies were not included because they were published before laparoscopic era or they were open bariatric studies. Also bariatric studies, which were published in other languages, were not searched and not included. This indicates a certain degree of selection bias. However, efforts were made to include the most powerful and robust study where possible.

5. Algorithm tables for management pathway is based on the evidence from these papers, majority of which were published in the last 3 years (2010–2013). It provides general management route and by no means represents a pathway for managing every case. Every patient has to be taken as an individual case.

6. Level of majority of evidence was L2+ (except in some areas), and this has to be taken in consideration when applying evidence in clinical practice. Surgeons, medical colleagues and emergency physicians practices and experience in dealing with bariatric patients have been evolving and the current view in this paper may help towards formulating guidelines to improve the standards of care these patients currently receiving, and avoid the preventable mortality and severe morbidity that unfortunately happening.

## Conclusions

Preventable serious complications can follow bariatric surgery, the effective management of which is depending on the awareness and early diagnosis of the acute surgical or medical cause. Effective communication with the bariatric team is essential to initiate an early and active management plan. Catastrophic outcomes are still reported and an algorithm of actions is proposed to prevent or reduce these incidents and mortality associated with bariatric emergencies.

## Competing interests

Dr AH and Dr SE have no conflicts of interest or financial ties to disclose.

## Author’s contribution

Dr AH designed the study, drafted the manuscript, critically appraised the paper and approved the final version. Dr SE contributed to the design and concept of the study, critically reviewed and approved the final version.
